# Alterations in lipid, redox, and energy metabolism in recent onset psychosis: a metabolomics study in non-smoking individuals and matched controls

**DOI:** 10.1016/j.redox.2025.103999

**Published:** 2025-12-26

**Authors:** Daphne A.M. Dielemans, Arjen L. Sutterland, René Lutter, Michel van Weeghel, Arno R. Bourgonje, Hanno L. Tan, Harry van Goor, Anja Lok, Nico J.M. van Beveren, Lieuwe de Haan, Julia M. Hagen

**Affiliations:** a Parnassia Academy, Parnassia Psychiatric Institute, The Hague, the Netherlands; b Department of Psychiatry, Amsterdam University Medical Center, University of Amsterdam, Amsterdam, the Netherlands; c Departments of Experimental Immunology and Respiratory Medicine, Amsterdam University Medical Center, University of Amsterdam, Amsterdam, the Netherlands; d Laboratory Genetic Metabolic Diseases, Core Facility Metabolomics, Amsterdam University Medical Center, University of Amsterdam, Amsterdam, the Netherlands; e Department of Gastroenterology and Hepatology, University Medical Center Groningen, University of Groningen, Groningen, the Netherlands; f The Dr. Henry D. Janowitz Division of Gastroenterology, Department of Medicine, Icahn School of Medicine at Mount Sinai, New York, United States; g Department of Experimental Cardiology, Amsterdam University Medical Center, University of Amsterdam, Amsterdam, the Netherlands; h Netherlands Heart Institute, Utrecht, the Netherlands; i Department of Pathology and Medical Biology, University Medical Center Groningen, University of Groningen, Groningen, the Netherlands; j Center for Urban Mental Health, University of Amsterdam, Amsterdam, the Netherlands; k Arkin Mental Health Institute, Amsterdam, the Netherlands

**Keywords:** Metabolomics, Schizophrenia spectrum disorder, Oxidative stress, Brain energy, Psychosis

## Abstract

Psychotic disorders are associated with systemic metabolic alterations, but these associations may be confounded by smoking. We investigated plasma metabolites in 47 non-smoking recent onset psychosis patients and 36 matched (on age, sex and ethnicity) healthy controls using untargeted LC MS metabolomics. We applied univariate, multivariate and pathway analyses, with subgroup exploration in patients that were diagnosed with schizophrenia spectrum disorder (SSD) specifically. We identified 28 significantly altered metabolites predominantly reflecting lipid metabolism (elevated saturated free fatty acids, glycerol 3 phosphate, conjugated bile acid), redox imbalances (decreased L cysteine, L cystine and taurine) and energy metabolism (reduced pyruvate). These alterations remained significant after adjusting for sex, antipsychotic treatment and metabolic syndrome parameters. Enrichment analyses highlighted taurine/hypotaurine metabolism, alanine/aspartate/glutamate pathways and fatty acid biosynthesis in psychosis. Within the SSD subgroup (n = 28), metabolic perturbations were more pronounced, showing stronger depletion of reducing equivalents and elevated free fatty acids. These findings indicate a specific systemic metabolic signature in psychosis independent of smoking, sex, antipsychotic medication or metabolic syndrome. The pattern suggests mitochondrial dysfunction and increased oxidative stress, accompanied by compensatory lipid mobilization. These findings identify redox and energy metabolism as promising targets for future pharmacological or metabolic interventions in SSD.

## Highlights

• Distinct plasma metabolomic signature found in psychosis, independent of smoking.
• Antioxidant metabolites such as cysteine and taurine were consistently reduced.
• Schizophrenia spectrum disorder subgroup showed more pronounced metabolic disturbances.
• Findings suggest mitochondrial dysfunction, oxidative stress, and a shift toward lipid mobilization.

## 1 Introduction

Schizophrenia and related psychotic disorders are severe mental illnesses characterized by positive symptoms (delusions, hallucinations), negative symptoms (affective flattening, loss of energy) and cognitive deficits. Despite the availability of antipsychotic treatments, many patients experience persistent symptoms and functional impairments [1]. This therapeutic gap underscores the continuing need to elucidate the biological mechanisms underlying psychosis, with the aim of improving early diagnosis, guiding stratified interventions, and identifying novel treatment targets. Multiple pathophysiological hypotheses have been proposed, including dopaminergic dysregulation, glutamatergic imbalance, neuroinflammation, and oxidative stress [2]. Oxidative stress refers to an imbalance between the production of reactive oxygen species (ROS) and the antioxidant defense systems that neutralize them, leading to damage of cellular macromolecules such as lipids, proteins, and DNA [3].

More recently, the bioenergetic dysfunction hypothesis has gained increasing attention. This hypothesis posits that brain function in schizophrenia and bipolar disorder is compromised due to altered cellular energy metabolism [4]. Deficits in glycolysis and the tricarboxylic acid (TCA) cycle, as well as impaired mitochondrial ATP production, have been reported in post-mortem and gene expression studies [5,6]. Mitochondrial dysfunction is a major source of ROS, directly linking bioenergetic deficits to oxidative stress. There is also a complex interplay between oxidative stress, inflammation, and glutamatergic signaling [7]. Disruption in any of these systems may propagate dysfunction across the others, potentially contributing to the widespread disturbances observed in schizophrenia and related psychotic disorders.

Growing evidence suggests that the pathophysiological disturbances in psychosis are not limited to the brain but are also reflected systemically. In particular, numerous studies have reported altered blood levels of oxidative stress and inflammatory markers in patients with first-episode psychosis (FEP), as well as in patients with chronic schizophrenia [[8], [9], [10]].

However, research into systemic alterations is difficult due to the lifestyle behavior of many individuals with psychosis. Especially oxidative stress is strongly influenced by smoking [11]. Besides oxidative stress, smoking also affects inflammation and metabolic regulation [12]. Given the high prevalence of smoking among patients with psychosis, it remains unclear whether systemic alterations in this population reflects the illness itself or the effects of smoking [8,9]. In individual studies, it is often difficult to control for smoking due to the small number of non-smoking participants. In meta-analyses, lack of access to individual participant data further impedes efforts to control for smoking. Beyond the limited ability to control for smoking, meta-analyses are also challenged by the diagnostic heterogeneity often present in psychosis cohorts, potentially obscuring disorder-specific biological effects [9].

We intended to collect a sample of individuals with recent onset psychosis (first psychosis less than 3 years ago) without any history of smoking. We investigated the plasma metabolome of these non-smoking cases and compared them to matched healthy controls. Specifically, we addressed the following research questions: (1) Is there a distinct plasma metabolomic profile that differentiates non-smoking patients with psychosis from non-smoking healthy controls? (2) Are the observed metabolic differences independent of other potentially confounding factors such as antipsychotic medication and metabolic syndrome variables? (3) Which metabolic pathways are represented by the metabolites that differ between psychotic patients and healthy controls? (4) Which metabolomic alterations are found in patients diagnosed with schizophrenia spectrum disorders, as a defined subgroup within the recent onset psychosis sample?

## 2 Materials and methods

### 2.1 Study participants

This study had a cross-sectional case-control design. Cases were recruited from the Department of Early Psychosis at Amsterdam UMC, location AMC, between 2012 and 2016. This department provides care to in- and outpatients with psychotic disorders, including both affective and non-affective diagnoses such as bipolar disorder (BD), schizophrenia, and psychosis not otherwise specified (NOS). All patients were in the early phase of psychosis treatment, defined as a duration of psychiatric care of less than three years. Exclusion criteria were a history of smoking and the presence of neurological disorders.

Controls were selected from the HELIUS (HEalthy LIfe in an Urban Setting) cohort, a population-based prospective study designed to investigate health disparities across ethnic groups. Detailed procedures have been described previously [13]. Participants were eligible as controls if they had no history of smoking, reported no psychiatric symptoms and did not use psychotropic medication.

Several demographic and clinical variables were collected for both groups. Demographic data included sex, age, educational level, alcohol consumption, and ethnicity. Metabolic syndrome parameters were assessed by physical examination (body mass index [BMI], waist circumference, blood pressure) and blood tests (fasting glucose, lipid profile). For cases, psychotic symptoms were evaluated using the Positive and Negative Syndrome Scale (PANSS) [14]. Information on duration of illness, use of medication, and psychiatric diagnosis according to the Diagnostic and Statistical Manual of Mental Disorders, Fourth Edition (DSM-IV) was obtained from the patients’ medical files.

Case-control matching was performed in a 1:1 ratio based on predefined variables: age (±3 years), and exact matching for sex and ethnicity.

### 2.2 Plasma sampling

Ethylenediaminetetraacetic acid (EDTA) plasma samples from both cases and controls were processed using identical procedures. Following venipuncture, samples were transported on ice and centrifuged within 1 h at 4 °C for 10 min. All samples were subsequently stored at −80 °C. Control samples were initially stored at −20 °C for a maximum of 24 h before being transferred to −80 °C.

### 2.3 Metabolite measurements

Metabolomics analysis was performed as previously described, with minor adjustments [15,16]. A 75 μL mixture of the following internal standards in water was added to 25 μL of plasma sample: D_7_-arginine (100 μM), ^13^C_3_-pyruvate (100 μM), D3-carnitine (100 μM), and D_5_-tryptophan (100 μM). Subsequently, 425 μL water, 500 μL methanol and 1 mL chloroform were added to the same 2 mL tube before thorough mixing and centrifugation for 10 min at 14,000 rpm. The top layer, containing the polar phase, was transferred to a new 1.5 mL tube and dried using a vacuum concentrator at 60 °C. Dried samples were reconstituted in 100 μL methanol/water (6/4, v/v). Metabolites were analyzed using a Waters Acquity ultra-high-performance liquid chromatography system (Waters Corporation, Milford, MA, USA) coupled to a Bruker Impact II™ Ultra-High Resolution Qq-Time-Of-Flight mass spectrometer (Bruker Daltonics, Billerica, MA, USA). Samples were kept at 12 °C during analysis and 5 μL of each sample was injected. Chromatographic separation was achieved using a Merck Millipore SeQuant ZIC-cHILIC column (PEEK 100 × 2.1 mm, 3 μm particle size). Column temperature was held at 30 °C. Mobile phase consisted of (A) 1:9 acetonitrile:water and (B) 9:1 acetonitrile:water, both containing 5 mM ammonium acetate. Using a flow rate of 0.25 mL/min, the LC gradient consisted of: Dwell at 100 % Solvent B, 0–2 min, Ramp to 54 % Solvent B at 13.5 min, Ramp to 0 % Solvent B at 13.51 min, Dwell at 0 % Solvent B, 13.51–19 min, Ramp to 100 % B at 19.01 min, Dwell at 100 % Solvent B, 19.01–19.5 min. Equilibrate the column using a 0.4 mL/min flow at 100 % B from 19.5 to 21 min. Mass spectrometry (MS) data were acquired using negative and positive ionization in full scan mode over the range of *m*/*z* 50–1200. Data were analyzed using Bruker Metaboscape software version 2024b.

All reported metabolite intensities were normalized to internal standards with comparable retention times and MS response. Metabolite identification was based on accurate mass, (relative) retention time (RT), and fragmentation spectra, compared against a library of authentic standards. Metabolites with both MS1 and RT data were considered confidently annotated (level 1), while those identified solely by MS1 spectra or database matches were classified as putative. A subset of unknown metabolites was structurally characterized using SmartFormula, based on isotopic patterns and fragmentation data. In some cases, metabolites with identical annotations but distinct retention times were detected. These were assigned suffixes to distinguish them. They likely represent isomers, isobars, or structurally related compounds, and were interpreted with caution.

### 2.4 Statistical analyses

Analyses were performed in RStudio (version 2024.09.0) using R version 4.4.2, with the following packages: xtable, DescTools, dplyr, tidyr, readxl, ggplot2, ggrepel, gridExtra, patchwork, purrr, randomForest, mixOmics, and ropls [[17], [18], [19], [20]].

The result of case-control matching was evaluated by comparing age, sex, and ethnicity between groups using independent samples t-tests (age) and chi-square tests (sex and ethnicity). Baseline characteristics were compared using t-tests, Mann-Whitney U tests, or Fisher's exact tests, depending on variable distribution and type.

### 2.5 Research question 1: metabolomic profiles in patients with psychosis vs. healthy controls

Group comparisons of metabolite levels were based on uncorrected peak height intensities and assessed using univariate tests. P-values were corrected for multiple testing using false discovery rate (FDR), Holm, and Bonferroni procedures. Metabolites were considered significant if FDR was <0.05. Fold-changes and log_2_-transformed fold changes were also calculated. Results from the negative and positive ionization modes were visualized using volcano plots to display statistical significance and fold-change. To explore group separation and identify discriminative metabolites, multivariate statistical methods were applied. Principal component analysis (PCA) was used for unsupervised dimensionality reduction and visualization of sample clustering. Supervised methods included partial least squares discriminant analysis (PLS-DA), orthogonal PLS-DA (OPLS-DA), and random forest classification. Variable importance in projection (VIP) scores were derived from PLS-DA models to assess the contribution of individual metabolites to group separation.

To aid the interpretation of metabolic profiles, significant annotation level 1 results from both positive and negative ionization modes were combined and categorized into functional groups. Based on prior literature and compound databases (PubChem, KEGG, HMDB), metabolites were grouped into three major metabolic categories: lipid metabolism, redox-related pathways and energy/carbohydrate metabolism [21,22]. Significantly altered metabolites that did not fall within these categories were classified as ‘other’, as they largely comprised degradation products, exogenous compounds, or intermediary metabolites with unclear or non-specific biological roles. If a metabolite was significantly different in both positive and negative ionization modes, the measurement from the mode with the most reliable quantification, as indicated by the lowest Δm/z and mSigma, was retained.

### 2.6 Research question 2: influence of confounding factors

To evaluate the influence of potential covariates on metabolite variation, additional analyses were conducted incorporating sex, alcohol consumption, use of antipsychotics and metabolic syndrome parameters (waist circumference, blood pressure, glucose, and triglyceride levels). The impact of these covariates on overall variance was assessed using PCA (cumulative Q^2^ calculation).

### 2.7 Research question 3: enriched pathways in patients with psychosis vs. healthy controls

For pathway analysis, we selected metabolites from the three main metabolic categories that showed significant case-control differences in univariate and multivariate analyses at annotation level 1. Pathway analysis was performed using MetaboAnalystR version 6.0 (https://www.metaboanalyst.ca/) with default settings and the human KEGG pathway library as reference. For each pathway, the analysis provided the number of observed and expected hits, raw and adjusted p-values (Holm and FDR), minus log10 p-values, and a pathway impact score based on relative betweenness centrality. Pathways were ranked by both statistical significance and biological relevance. Plots were used to reflect impact of altered metabolites on pathways, with pathways grouped into the three functional clusters and ordered by pathway-level significance. In addition, log_2_ fold changes from individual metabolites were used to visualize metabolite differences within enriched pathways.

### 2.8 Research question 4: metabolomic profiles of patients with schizophrenia spectrum disorder

Univariate, multivariate and pathway analyses were also repeated in the subsample of recent onset psychosis patients whose psychosis was classified at examination under the diagnostic categories of schizophrenia, schizophreniform, or schizoaffective disorder (according to DSM-IV criteria). These cases were categorized as having a schizophrenia spectrum disorder (SSD), and their matched controls were also selected from the full sample to examine subgroup-specific effects. This distinction was made because recent onset and FEP populations are known to be heterogeneous, and distinct biological mechanisms are likely to underlie other diagnostic subtypes within the broader psychosis spectrum [9].

### 2.9 Ethical considerations

The study protocol was reviewed and approved by the Institutional Review Board of Amsterdam UMC (Medical Ethics Committee, METC). Written informed consent was obtained from all participants. The study was conducted in accordance with the Declaration of Helsinki (version 9-10-2004) and the Medical Research Involving Human Subjects Act (WMO).

## 3 Results

### 3.1 Descriptive characteristics of participants

Forty-seven individuals with psychosis and 36 matched controls were enrolled. None of the participants ever smoked. Demographic and clinical characteristics are summarized in Table 1. Age, sex and ethnicity did not differ between groups (all p > 0.05), confirming successful matching. A detailed breakdown of diagnostic categories is provided in Supplementary Table 1.

**Table 1:** Descriptive characteristics of study participants.

		Cases (n = 47)		Controls (n = 36)		p-value
Age (years)	mean (SD)	24.55	(3.80)	24.61	(3.67)	0.94
**Sex**						
Female	n (%)	17	(36.17)	20	(55.56)	0.12
Male	n (%)	30	(63.83)	16	(44.44)	0.12
**Ethnicity**						
- European	n (%)	26	(55.32)	18	(50.00)	0.66
- Northern African	n (%)	6	(12.77)	6	(16.67)	0.76
- Sub-Saharan	n (%)	2	(4.26)	2	(5.56)	1
- Surinamese and Antillean	n (%)	4	(8.51)	6	(16.67)	0.32
- Turkish	n (%)	3	(6.38)	4	(11.11)	0.46
- Unknown	n (%)	6	(12.77)	0	0	0.03
**Educational level**						
- Higher	n (%)	14	(29.79)	15	(41.67)	0.35
- Secondary	n (%)	16	(34.04)	15	(41.67)	0.5
- Lower secondary	n (%)	14	(29.79)	4	(11.11)	0.06
- Primary	n (%)	2	(4.26)	1	(2.78)	1
**Substance use**						
Alcohol consumption (yes)	n (%)	23	(48.94)	19	(52.78)	0.83
**Metabolic Parameters**						
BMI (kg/m2)	mean (SD)	24.31	(6.15)	23.59	(5.02)	0.58
Blood total cholesterol (mmol/L)	mean (SD)	4.49	(0.82)	4.2	(0.75)	0.12
Blood HDL cholesterol (mmol/L)	mean (SD)	1.4	(0.36)	1.44	(0.36)	0.66
Blood triglycerides (mmol/L)	mean (SD)	0.99	(0.56)	0.71	(0.28)	0.01
Use of antihyperlipidemic medication	n (%)	0	0	0	0	
Blood glucose (mmol/L)	mean (SD)	5.26	(0.74)	4.96	(0.36)	0.02
Use of antidiabetic medication	n (%)	0	0	0	0	
Systolic blood pressure (mmHg)	mean (SD)	122.77	(15.11)	121.29	(14.21)	0.66
Diastolic blood pressure (mmHg)	mean (SD)	78.41	(10.58)	73.28	(9.21)	0.03
Use of antihypertensives	n (%)	1	(2.17)	0	0	1
Waist circumference (cm)	mean (SD)	90.52	(17.89)	80.02	(13.76)	0.02
Metabolic syndrome	n (%)	3	(13.04)	1	(2.78)	0.29
**Diagnosis**						
- Schizophrenia spectrum disorder	n (%)	28	(59.57)			
- Bipolar disorder	n (%)	9	(19.15)			
- Other	n (%)	10	(21.28)			
**Disease related parameters**						
Duration of illness (years)	median (IQR)	0.58	(0.72)			
Total PANSS score	mean (SD)	64.84	(23.39)			
PANSS positive score	mean (SD)	14.67	(6.73)			
PANSS negative score	mean (SD)	16.64	(8.21)			
Use of antipsychotic medication	n (%)	24	(51.00)			
Antipsychotic dosage (olanzapine equivalent in mg)	median (IQR)	6.66	(10.94)			

Table 1. Demographic, clinical and metabolic characteristics of psychosis cases and healthy controls. The table summarizes age, sex, ethnicity, educational level, substance use, and a range of metabolic parameters for all participants. Disease-related characteristics including psychiatric diagnosis, illness duration, PANSS scores and use of antipsychotic medication are presented for the patient group only. Depending on the distribution and type of variable, data are presented as mean (SD), median (IQR), or counts with percentages. Corresponding p-values reflect between-group comparisons based on appropriate statistical tests (e.g., t-tests, Mann–Whitney U tests, or χ^2^ tests).

### 3.2 Plasma metabolomic profile in patients with psychosis vs. healthy controls

#### 3.2.1 Univariate analyses

We identified 28 metabolites that differed significantly (FDR < 0.05) between psychotic patients and healthy controls. Out of this total, 21 mapped to three metabolic clusters (lipid metabolism, redox-related pathways, and energy and carbohydrate metabolism) and 7 metabolites belonged to other metabolic pathways. An overview of the 21 cluster-related metabolites is provided in Table 2. Notable increases were observed in lipid-related metabolites, including glycerol-3-phosphate and conjugated bile acids. Glycolytic sugars were significantly increased, although these signals may also represent other isomeric hexoses indistinguishable by MS. Most decreased metabolites were related to redox regulation or central carbon metabolism, including l-cysteine, l-cystine, l-methionine, taurine, bilirubin, gamma-glutamylglutamine, and pyruvic acid. Complete statistics for all 28 identified significant metabolites are provided in Supplementary Table 2. Supplementary Fig. 1 displays volcano plots for both ionization modes.

**Table 2:** Overview of metabolites associated with psychosis.

Metabolite	Cluster	Function	Mean peak insensity		Fold Change	Log2 Fold Change	FDR	VIP-score
			Cases	Controls				
***Increased in cases***								
Palmitic acid	Lipids and lipid-related metabolites	Storage and membrane lipids (free fatty acids)	1,46,58,883.83	1,36,55,271.78	1.07	0.10	0.000	2.60
Stearic acid	Lipids and lipid-related metabolites	Storage and membrane lipids (free fatty acids)	1,27,05,350.89	1,09,39,056.17	1.16	0.22	0.000	2.48
Capric acid	Lipids and lipid-related metabolites	Storage and membrane lipids (free fatty acids)	3,70,433.53	2,81,107.83	1.32	0.40	0.001	1.98
Glycerol 3-phosphate	Lipids and lipid-related metabolites	Lipid-metabolic related metabolites (glycerolipid intermediate)	1,85,386.17	98,390.89	1.88	0.91	0.001	2.01
Chenodeoxycholic acid glycine conjugate	Lipids and lipid-related metabolites	Storage and membrane lipids (bile acids)	27,549.57	11,494.11	2.40	1.26	0.002	1.86
Ascorbic acid	Redox-related	Antioxidants & ROS buffers (ROS scavenger)	30,651.11	23,640.56	1.30	0.37	0.002	2.01
d-Mannose	Energy & carbohydrate metabolism	Glycolysis	1,05,153.91	87,714.61	1.20	0.26	0.005	1.96
l-Aspartic acid	Energy & carbohydrate metabolism	TCA cycle and anaplerosis	22,632.47	18,343.67	1.23	0.30	0.017	1.74
2-Deoxyglucose	Energy & carbohydrate metabolism	Glycolysis	24,569.23	20,030	1.23	0.29	0.036	1.49

***Decreased in cases***								
Pyruvic acid	Energy & carbohydrate metabolism	Glycolysis	1,55,384.04	2,15,187.11	0.72	−0.47	0.000	2.36
l-Cysteine	Redox-related	Gluthatione cycle (glutathione precursor)	7000.13	11,544.06	0.61	−0.72	0.000	2.27
Taurine	Redox-related	Antioxidants & ROS buffers (ROS buffer)	3,67,164.09	5,27,379.39	0.70	−0.52	0.000	2.29
l-Cystine	Redox-related	Gluthatione cycle (glutathione precursor)	6857.66	11,175.00	0.61	−0.70	0.001	2.21
Stachyose	Energy & carbohydrate metabolism	Glycan & sugar derivatives	2022.38	3325.44	0.61	−0.72	0.001	2.12
N-Acetylneuraminic acid	Energy & carbohydrate metabolism	Glycan & sugar derivatives	3810.94	5035.94	0.76	−0.40	0.002	1.89
l-Glutamine	Energy & carbohydrate metabolism	TCA cycle and anaplerosis	39,111.57	53,288.44	0.73	−0.45	0.002	2.11
Pelargonic acid	Lipids and lipid-related metabolites	Storage and membrane lipids (free fatty acids)	45,577.91	48,572.61	0.94	−0.09	0.002	2.05
gamma-Glutamylglutamine	Redox-related	Gluthatione cycle (glutathione precursor)	57,335.36	74,399.33	0.77	−0.38	0.006	2.01
N-Acetyl-l-aspartic acid	Energy & carbohydrate metabolism	TCA cycle and anaplerosis	19,014.21	91,761.00	0.21	−2.27	0.011	2.03
Raffinose	Energy & carbohydrate metabolism	Glycan & sugar derivatives	5588.98	8778.83	0.64	−0.65	0.011	1.72
Bilirubin	Redox-related	Antioxidants & ROS buffers (lipophilic antioxidant)	11,394.34	18,011.67	0.63	−0.66	0.012	1.67

Table 2. Summary of 21 key metabolites and their corresponding functional clusters. The table reports mean peak intensities for cases and controls, fold changes, false discovery rates (FDR), and variable importance in projection (VIP) scores, sorted by FDR.

#### 3.2.2 Multivariate analyses

An unsupervised PCA of all annotated metabolites revealed partial clustering of psychotic patients and healthy controls (Supplementary Fig. 2). The first two principal components captured a substantial proportion of total variance (25.8 % in positive-ion mode, 22.3 % in negative-ion mode) and tended to separate the groups. VIP scores derived from the PLS-DA models are reported in Table 2, alongside the fold changes and FDR-values. Metabolites with VIP values greater than 2 included palmitic acid, stearic acid, pyruvic acid, stachyose, the cysteine/cystine pair, and taurine, many of which also showed large fold changes and highly significant p-values. Detailed performance metrics for both the OPLS-DA and the random forest models are provided in Supplementary Table 3.

### 3.3 Influence of confounding factors

To assess the influence of potential confounding factors, we regressed out sex, antipsychotic dosage, alcohol consumption, waist circumference, diastolic blood pressure, fasting glucose, and blood triglyceride levels. The resulting change in cumulative Q^2^ was minimal (≤0.030 in positive-ion mode, ≤0.006 in negative-ion mode). After adjustment for sex, most metabolites showed comparable or higher VIP scores compared to the unadjusted models. Only pelargonic acid showed a marked reduction in VIP score, suggesting that its contribution to the model was more strongly affected by sex. When VIP scores were compared after regressing out antipsychotic dosage alone, all metabolites showed consistent associations. However, when adjusting for metabolic parameters, not all results remained robust. Notably, VIP scores for ascorbic acid and 2-deoxyglucose decreased substantially. Hexose (probably d-mannose), glycocholic acid, l-glutamine, pelargonic acid, glycerol 3-phosphate, and l-carnitine also appeared to be more strongly influenced by these confounders than by diagnostic status alone. Full results are presented in Supplementary Table 4.

### 3.4 Differences in metabolic pathways between psychosis patients and healthy controls

**Fig. 1:**
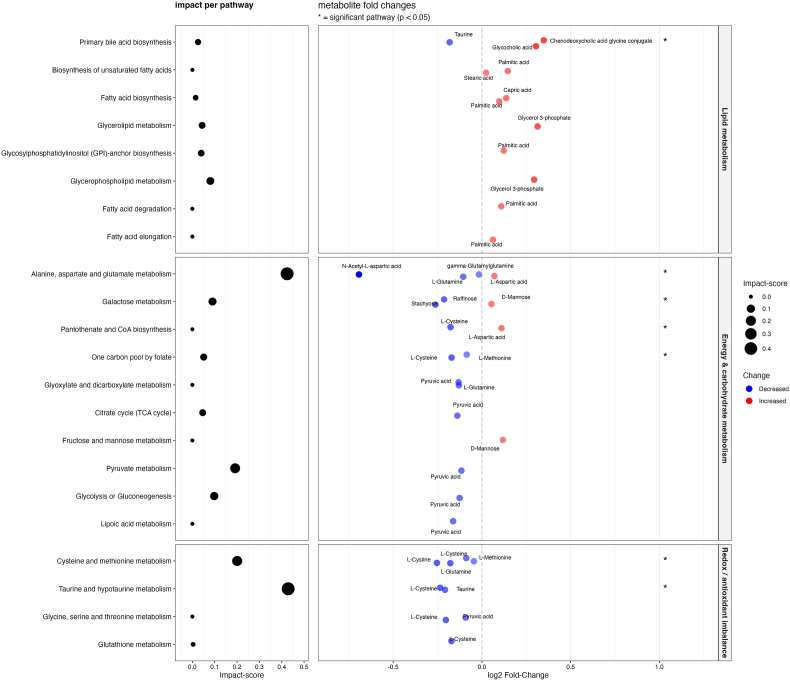
Pathway analysis: full sample Pathway analysis of differentially expressed metabolites between psychosis cases and controls. Enriched metabolic pathways are grouped into lipid metabolism (top panel), energy and carbohydrate metabolism (middle panel), and redox/antioxidant-related functions (bottom panel). The left panels display pathway impact scores, with dot size corresponding to each pathway's relative impact. The right panels show individual metabolite fold changes on a log_2_ scale. Red dots represent metabolites increased in cases, blue dots indicate decreases and color intensity reflects the magnitude of change. Significantly altered pathways are indicated by an asterisk (∗).

A total of 23 pathways could be mapped to one of the three earlier recognized functional clusters. Pathway analysis revealed significant enrichment (p < 0.05) in 6 pathways: galactose metabolism, alanine, aspartate and glutamate metabolism, primary bile acid biosynthesis, pantothenate and CoA biosynthesis, one carbon pool by folate, cysteine and methionine metabolism, and taurine and hypotaurine metabolism (Fig. 1). The pathways with highest cumulative impact scores were related to redox imbalance and energy metabolism. Among metabolites driving enrichment, pyruvic acid, l-cysteine and glycerol-3-phosphate showed strong contributions across multiple enriched pathways. Detailed pathway enrichment statistics are available in Supplementary Table 5.

### 3.5 Metabolomic profiles of patients with SSD

In the SSD subgroup (n= 28), 16 metabolites differed significantly from matched controls. Findings largely overlapped with those observed in the full sample, with several effects appearing more pronounced. Most elevated metabolites were lipid-related, including capric acid (C10:0), stearic acid (C18:0), and palmitic acid (C16:0). Decreased metabolites were primarily related to redox and energy metabolism, including l-cysteine, l-cystine, and pyruvic acid. Isobutyrylglycine was significantly elevated in this subgroup but not in the full sample. Univariate results and VIP-scores are summarized in Table 3 and detailed outcomes are presented in Supplementary Table 6. Supplementary Fig. 3 displays volcano plots for the SSD subgroup. Multivariate analysis results (PCA, OPLS-DA and random forest classification) are provided in Supplementary Fig. 4 and Supplementary Table 7.

**Table 3:** Overview of altered metabolites in schizophrenia spectrum disorder subgroup.

Metabolite	Cluster	Function	Mean peak insensity		Fold Change	Log2 Fold Change	FDR	VIP-score
			Cases	Controls				
***Increased in cases***								
Palmitic acid	Lipids and lipid-related metabolites	Storage and membrane lipids (free fatty acids)	1,47,41,646.79	1,36,19,904.88	1.08	0.11	0.001	2.66
Stearic acid	Lipids and lipid-related metabolites	Storage and membrane lipids (free fatty acids)	1,28,54,837.50	1,09,18,057.68	1.18	0.24	0.002	2.43
Glycerol 3-phosphate	Lipids and lipid-related metabolites	Lipid-metabolic related metabolites (glycerolipid intermediate)	1,89,965.14	96,960.08	1.96	0.97	0.015	2.02
Isobutyrylglycine	Lipids and lipid-related metabolites	Lipid-metabolic related metabolites (acylglycines)	5311.43	3904.64	1.36	0.44	0.019	1.95
Capric acid	Lipids and lipid-related metabolites	Storage and membrane lipids (free fatty acids)	3,71,808.00	2,74,062.16	1.36	0.44	0.028	1.84
Ascorbic acid	Redox-related	Antioxidants & ROS buffers (ROS scavenger)	30,042.86	22,817.60	1.32	0.40	0.028	1.91
Chenodeoxycholic acid glycine conjugate	Lipids and lipid-related metabolites	Storage and membrane lipids (bile acids)	29,358.79	12,304.72	2.39	1.25	0.043	1.72

***Decreased in cases***								
l-Cysteine	Redox-related	Gluthatione cycle (glutathione precursor)	6486.36	12,041.92	0.54	−0.89	0.002	2.50
l-Cystine	Redox-related	Gluthatione cycle (glutathione precursor)	6707.57	11,846.88	0.57	−0.82	0.003	2.42
Stachyose	Energy & carbohydrate metabolism	Glycan & sugar derivatives	1826.07	3225.52	0.57	−0.82	0.014	2.21
Pyruvic acid	Energy & carbohydrate metabolism	Glycolysis	1,57,097.71	2,10,841.52	0.75	−0.42	0.014	2.04
Taurine	Redox-related	Antioxidants & ROS buffers (ROS buffer)	3,55,709.71	5,20,570.16	0.68	−0.55	0.014	2.13
Pelargonic acid	Lipids and lipid-related metabolites	Storage and membrane lipids (free fatty acids)	45,815.00	49,121.36	0.93	−0.10	0.025	1.95
N-Acetyl-l-aspartic acid	Energy & carbohydrate metabolism	TCA cycle and anaplerosis	21,012.57	1,07,596.48	0.20	−2.36	0.038	1.97
Raffinose	Energy & carbohydrate metabolism	Glycan & sugar derivatives	4986.50	8051.20	0.62	−0.69	0.038	1.84
N-Acetylneuraminic acid	Energy & carbohydrate metabolism	Glycan & sugar derivatives	3668.71	4955.20	0.74	−0.43	0.043	1.72

Table 3. Results for the schizophrenia spectrum disorder subgroup, including mean peak intensities for cases and their matched controls, fold changes, false discovery rates (FDR), and variable importance in projection (VIP) scores. Results are sorted by FDR.

**Fig. 2:**
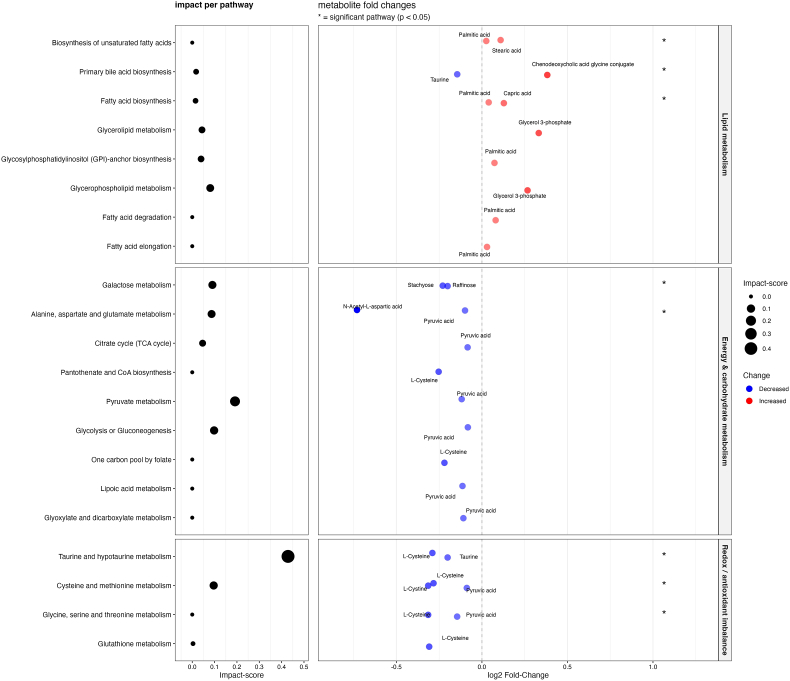
Pathway analysis: subgroup with schizophrenia spectrum disorder Pathway analysis of differentially expressed metabolites in the schizophrenia spectrum disorder (SSD) subgroup compared to matched controls. The visualization follows the same structure as in Fig. 1. The overall pattern was largely consistent with that of the full sample, with increased metabolites clustering in lipid metabolism and reductions observed in energy and redox-related pathways.

The pathway enrichment analysis results for the SSD subgroup are visualized in Fig. 2 and detailed outcomes are presented in Supplementary Table 8. In the SSD subgroup, lipid-related pathways were prominently affected, with significant enrichment particularly in fatty acid biosynthesis. Redox-related and energy metabolism pathways also showed marked deviations. The glycine, serine and threonine metabolism, pantothenate and CoA biosynthesis and one carbon pool by folate pathway were significantly altered in the full sample, but did not reach statistical significance in the SSD subgroup.

## 4 Discussion

In the current study, we investigated plasma metabolites in non-smoking individuals with recent onset psychotic disorders compared to non-smoking healthy controls. The main alterations were characterized by elevated lipid-related metabolites alongside reductions in compounds involved in redox regulation and energy metabolism. Our study provides evidence of smoking-independent systemic metabolic alterations in patients with a psychotic disorder, which are associated with oxidative stress and mitochondrial dysfunction. Apart from smoking, we accounted for several other key confounders, including sex, antipsychotic medication use and metabolic parameters. The findings were consistent across both the full cohort and the SSD subgroup, with some effects appearing more pronounced in the latter.

By integrating comprehensive metabolic profiling with the deliberate exclusion of smokers, our study offers a unique contribution to both metabolomics and psychosis research. Several metabolomics studies in psychosis did not account for smoking status, a limitation that may bias observed metabolic patterns, for example in antioxidants, bile acids, and acylcarnitines [10,[23], [24], [25], [26]]. Smoking is a major source of systemic oxidative stress, leading to reduced antioxidant defenses, increased lipid peroxidation, and oxidative protein modifications, thereby influencing a wide range of metabolic pathways [11,27,28]. Studies that did consider smoking generally restricted their analyses to specific metabolic domains, such as lipids or amino acids, providing only a partial view of systemic metabolic alterations [[29], [30], [31]]. By excluding smokers and applying a broad metabolomics approach, our study provides greater certainty of metabolic alterations are attributable to psychosis rather than to smoking-related effects.

Perturbations in antioxidant metabolites, such as glutathione, have been consistently observed in psychosis [32]. In the present study, we found several indications of redox imbalance and we were able to demonstrate that these alterations occurred independently of cigarette smoking. Glutathione precursors l-cysteine and l-cystine were reduced, suggesting depleted antioxidant reserves [21,23,29]. Taurine and bilirubin, both antioxidants, were also decreased. While studies have reported mixed findings on taurine, our results align with increased taurine consumption under oxidative stress [30,31,33]. No evidence supported impaired taurine synthesis, and the taurine/hypotaurine pathway was enriched in both full sample and SSD subgroup. Importantly, taurine and bilirubin remained significantly lower after adjustment for metabolic parameters, suggesting that these alterations are not solely driven by metabolic syndrome. Redox-related metabolite gamma-glutamyl glutamate showed alterations in the full sample, but did not replicate in subgroup analyses. Gamma-glutamyl peptides are produced during glutathione catabolism via the activity of glutathione transferases [34]. While some studies reported lower gamma-glutamyl peptide levels in SSD, our inability to replicate this in the SSD subgroup suggests heterogeneity [34,35].

Alterations in amino acid profiles in psychosis are generally considered secondary to metabolic or oxidative dysregulation, given the role of amino acids in the TCA cycle and gluconeogenesis. l-glutamine reduction is among the most consistently reported alterations, observed across both medicated and unmedicated psychotic individuals, and similarly detected in our study [30,33,36]. Glutamine is central to the alanine, aspartate, and glutamate metabolism pathway, which was significantly enriched in both the full recent onset psychosis cohort and the SSD subgroup, consistent with previous research [37]. l-aspartic acid was elevated in the full sample, but not in the SSD subgroup. This aligns with previously inconsistent findings and may reflect clinical heterogeneity across study populations [24,37,38]. The neuronal derivative of l-aspartic acid, N-acetylaspartate (NAA), was significantly reduced in our study. Plasma NAA reductions are particularly found in younger SSD populations [21]. However, the relationship between plasma and brain NAA levels is unclear [39,40].

N-acetylneuraminic acid (NaNeu, sialic acid) was also decreased, consistent with evidence from platelets [41], post-mortem tissue [40] and cerebrospinal fluid [42]. NaNeu is the terminal component of many glycoproteins and glycolipids and is essential for the synthesis of polysialylated neural cell adhesion molecules (PSA-NCAMs). PSA-NCAMs have been found to be dysregulated in the prefrontal cortex in SSD [43]. Although reduced peripheral NaNeu may reflect structural or neurodevelopmental disruption, it is more likely to result from a compensatory shift toward lipid-based synthesis to support sphingolipid production under oxidative stress. Lipids are highly vulnerable to oxidative damage, which can alter membrane fluidity, receptor function, and cellular signaling [44]. Consequently, alterations in structural and signaling lipids, including phospholipids, sphingolipids, and eicosanoids have been found in SSD [45,46]. Because mitochondrial membranes are lipid-rich, oxidative lipid damage may further compromise energy production, creating a self-reinforcing cycle of metabolic dysfunction [5]. Lipolysis is therefore hypothesized as a compensatory mechanism for the impaired energy metabolism in SSD [47].

Findings on saturated FFA levels in SSD have been inconsistent, with some studies reporting increases in palmitic and stearic acids and others reporting decreases [21,48,49]. Xuan et al. observed lower saturated FFA levels in risperidone-treated patients, leading to suggestions that antipsychotic treatment may normalize these metabolites [38]. Our findings challenge this interpretation, as elevated levels of palmitic and stearic acids persisted even after adjusting for antipsychotic medication and metabolic parameters. Instead, our findings support the view that increased FFA levels may result from enhanced lipolysis and β-oxidation [48]. Pathway enrichment analysis also confirmed alterations in the biosynthesis of lipids in SSD.

Glycerol-3-phosphate (G3P) forms a central link between the carbohydrate and lipid metabolism. During lipolysis, glycerol is converted to G3P while FFAs are released. Within the G3P shuttle, mitochondrial glycerol-3-phosphate dehydrogenase (mGPDH) oxidizes G3P to dihydroxyacetone phosphate (DHAP) and contributes to oxidative phosphorylation. Notably, mGPDH is a major source of ROS production [50]. G3P elevation, together with a near-significant DHAP reduction, indicates disruption of this pathway. Clozapine can activate the cytosolic GPDH isoform (cGPDH), driving DHAP-to-G3P conversion [51]. Although thirteen participants were receiving clozapine in our study, the elevation in G3P remained after accounting for medication effects, which may indicate underlying mitochondrial dysfunction and increased oxidative stress. Pyruvate was also reduced, further pointing towards a decreased mitochondrial uptake [4].

Several metabolites of dietary origin were decreased in patients, including raffinose, stachyose, and pelargonic acid. Raffinose and stachyose are fermented by intestinal microbiota. Lower plasma levels may reflect dietary differences or altered gut microbial activity, supported by galactose metabolism pathway enrichment. This aligns with increasing evidence of gut microbiome disruption in SSD [52]. Interestingly, prior work has linked altered pelargonic acid levels to brain-gut axis disturbances [47]. d-mannose is also primarily of dietary origin and increased levels are associated with a higher risk of type 2 diabetes and cardiovascular disease [53]. These findings indicate that elevated mannose may reflect a broader metabolic susceptibility in psychosis. This interpretation is further supported by the feature annotated as 2-deoxyglucose, which showed elevation in the full recent onset psychosis cohort, which was sensitive to metabolic correction and not present in the SSD subgroup. The observed alterations in glucose metabolism are particularly notable given the increased prevalence of type 2 diabetes among individuals with schizophrenia spectrum disorders and the proposed presence of underlying metabolic disturbances associated with the illness [54]. It is worth noting that the identified sugars may represent different hexose isomers, as mass spectrometry alone cannot reliably distinguish between such structurally similar compounds. Putative annotations such as 2-deoxyglucose, stachyose or mannose may therefore reflect other (deoxy)hexose- or oligosaccharide-related features and should be interpreted with caution.

Last, we observed elevated conjugated bile acids, particularly glycine-conjugated forms. Prior studies already reported disruptions in bile acid homeostasis in SSD, but these studies did not account for confounding factors [55,56]. Chenodeoxycholic acid glycine conjugate remained robustly elevated after correction for metabolic covariates and medication status. Our pathway enrichment analysis further confirmed involvement of primary bile acid biosynthesis. The concurrent decrease in taurine suggests a shift from taurine-to glycine-conjugated bile acids, potentially as a result of oxidative stress [57]. Of note, due to the presence of multiple isomeric or isobaric compounds, any interpretation of these conjugated bile acids should be considered tentative.

Taken together, our findings reveal impaired energy metabolism and oxidative stress in recent onset psychotic disorders (including SSD), accompanied by a shift towards lipolysis and β-oxidation as compensatory mechanisms. These alterations are not only biologically meaningful but also clinically relevant, as they point to potentially modifiable targets. This highlights the potential for interventions aimed at restoring metabolic balance by targeting these disrupted pathways through pharmacological or nutritional compounds with antioxidant or mitochondrial-stabilizing properties. For instance, N-acetylcysteine has shown promise in reducing oxidative stress by increasing glutathione and improving clinical outcomes in individuals with SSD [58]. Other agents with antioxidant or lipid-modulating properties, such as omega-3 fatty acids and polyunsaturated fatty acids (PUFAs), have also been suggested as potentially effective interventions in psychosis [59]. Moreover, metabolic substrates such as ketone bodies may help restore energy balance by bypassing impaired glucose metabolism and supporting mitochondrial and neuronal function through reduced oxidative stress [60].

### 4.1 Strengths and limitations

This study has several strengths. First, the well-matched case-control design, including careful consideration of ethnic diversity, reduces the likelihood of confounding due to unmeasured demographic factors. Second, by explicitly accounting for heterogeneity within the recent onset psychosis group, the analysis mitigates the risk that clinically relevant metabolic alterations are masked by diagnostic variability. Interestingly, metabolites not primarily associated with SSD showed the greatest changes in association after adjustment for confounders, underscoring the substantial impact of these variables on observed group differences. Moreover, the study identified metabolites whose alterations appear to be related to SSD itself, rather than to medication use or other secondary influences. By focusing on a relatively young cohort, the study further reduces the likelihood of secondary effects related to chronicity, treatment duration, or disease complications, allowing for a clearer view of early pathophysiological processes. Some limitations must also be acknowledged. The findings are based on peripheral blood measurements and therefore do not provide direct insight into metabolite concentrations or processes within the central nervous system or cerebrospinal fluid. Additionally, the application of extensive correction for multiple comparisons, while necessary to control false positives, may have reduced sensitivity to detect smaller, but potentially relevant effects. Last, the relatively small sample size and recruitment from a single clinical center may limit the generalizability of our findings.

## 5 Conclusion

In conclusion, current findings collectively point to a systemic metabolic phenotype in psychosis marked by redox imbalance, mitochondrial dysfunction, and compensatory lipid mobilization. The persistence of key alterations after adjustment for antipsychotic medication and metabolic covariates supports the notion that these shifts are reflective of underlying pathophysiological processes rather than secondary treatment effects. The attenuation of several findings in subgroup analyses further highlights the importance of accounting for diagnostic heterogeneity and individual variation in future research. To build on these findings, future research should aim to elucidate the mechanistic origins of these metabolic shifts by linking peripheral alterations to central processes. This could be achieved by incorporating cerebrospinal fluid sampling or neuroimaging techniques. Importantly, a better understanding of these mechanisms may guide the development of novel treatment strategies that specifically target the affected pathways.

## CRediT authorship contribution statement

**Daphne A.M. Dielemans:** Data curation, Formal analysis, Writing – original draft. **Arjen L. Sutterland:** Conceptualization, Investigation, Methodology, Resources, Writing – review & editing. **René Lutter:** Conceptualization, Methodology, Resources, Validation, Visualization, Writing – review & editing. **Michel van Weeghel:** Methodology, Resources, Validation, Writing – review & editing. **Arno R. Bourgonje:** Writing – review & editing. **Hanno L. Tan:** Writing – review & editing. **Harry van Goor:** Writing – review & editing. **Anja Lok:** Conceptualization, Resources, Writing – review & editing. **Nico J.M. van Beveren:** Supervision, Writing – review & editing. **Lieuwe de Haan:** Conceptualization, Funding acquisition, Supervision, Writing – review & editing. **Julia M. Hagen:** Conceptualization, Data curation, Funding acquisition, Methodology, Project administration, Supervision, Writing – review & editing.

## Declaration of competing interest

Arno R. Bourgonje reports receiving research grants from Janssen Pharmaceuticals and received speaker's fees from AbbVie and Ferring Pharmaceuticals, outside the submitted work.

All other authors declare no conflicts of interest.

## Data Availability

Data will be made available on request.
